# Spatio-temporal epidemiology of Japanese encephalitis in Nepal, 2007-2015

**DOI:** 10.1371/journal.pone.0180591

**Published:** 2017-07-26

**Authors:** Dhan Kumar Pant, Tenzin Tenzin, Rakesh Chand, Barun Kumar Sharma, Padam Raj Bist

**Affiliations:** 1 Institute of Medicine, Tribhuvan University, Kathmandu, Nepal; 2 National Zoonoses and Food Hygiene Research Centre, Kathmandu, Nepal; 3 National Centre for Animal Health, Thimphu, Bhutan; 4 Ministry of Livestock Development, Government of Nepal, Kathmandu, Nepal; University of Texas Medical Branch at Galveston, UNITED STATES

## Abstract

Japanese encephalitis (JE) is a major public health problem in Nepal. For the effective management and surveillance of JE, a clear understanding of its epidemiology is essential. Therefore, we conducted descriptive and spatial analyses to understand the spatio-temporal distribution of JE in human in Nepal. From 2007 to 2015, 1,823 JE cases were reported with a cumulative mean incidence of 0.735/100,000 population and a case fatality rate of 6.6%. The death rate in the up-to-24 years of age group was 74%. The JE cases were most commonly reported in the age group of 1–14 years. There is a strong seasonal pattern of JE occurrence in Nepal which peaked in August and declined by October each year, which corresponds to the monsoon season. The JE cases were reported in 63 of 75 districts (84%), expanding in the mountain and hill regions. There was a strong clustering of JE incidence in the south-western and south-eastern Terai region, which is endemic for JE. Therefore, the JE surveillance system should be improved to better understand the drivers of disease expansion in Nepal for instituting a control program.

## Introduction

Japanese encephalitis (JE) is a vector-borne viral disease caused by a *flavivirus*. The transmission cycle typically involves *Culex tritaeniorhynchus* mosquitoes and similar species, with aquatic birds and pigs acting as reservoir and amplifying hosts, respectively. Humans are the incidental host and play no role in perpetuating the virus [1]. The first epidemic of JE was reported in Japan during 1924 [1,2]. Thereafter, the epidemiological trend has been increasing in Asian countries, particularly in South East Asia [3]. In recent years, the epidemiological patterns and distribution of JE have changed. The disease is spreading in wider geographical areas due to climate change, land use patterns (especially cultivation of land and urbanization), changes in socioeconomic status (promotion of pig breeding as food source) and vector diversity [4–6]. Globally, approximately 3 billion people live in JE risk areas and nearly 68,000 clinical cases of JE, with 13,600 to 20,400 deaths, occur each year [7]. The case fatality rate of JE ranges from 10–20%, while 20–30% of the survivors have permanent neurological disorder [8]. Fundamentally, JE is considered as a disease of children, but all age groups are vulnerable in tropical and subtropical countries [3,8,9].

In Nepal, JE was first confirmed in the Rupandehi district during 1978 [10]. Since then, JE has been reported in other areas, with an occurrence of a large number of cases every 2 to 5 years [11]. Today, it is being reported from 63 of 75 districts, including the capital city area—Kathmandu valley [12,13]. Since 2004, Nepal began Japanese encephalitis virus (JEV) surveillance through a network of national vaccine preventable diseases, with technical and financial support from the World Health Organization (WHO) [14]. In addition, the Ministry of Health has implemented a phase-wise mass immunization programme in JE endemic districts, targeting children less than 15 years of age. This has resulted in a significant reduction of JE cases [11,13, 15]. However, for the effective management and surveillance of JEV, a clearer understanding of disease epidemiology is essential. In this study, we analyzed JEV surveillance data from 2007 to 2015, with an objective to identify the spatio-temporal pattern of reported JE cases in humans. The findings from this study would be useful in making policy decisions on improved JEV surveillance, prevention and control in Nepal.

## Methods

### Study area

Nepal is a landlocked mountainous country situated between China in the north and India in the south, east and west, with a human population of approximately 29 million [16]. Topographically, Nepal is divided into three distinct ecological regions: Mountain, Hills and Terai. The Terai region in the south is the grain basket of Nepal, with a high precipitation and high relative humidity, providing favorable environments for the breeding of mosquitoes (*C*. *tritaeniorrhynchus*), a proven vector of JE in Nepal [11].

### Current JE surveillance system

Three epidemiological surveillance and reporting systems exist for JEV in Nepal. They are: a health management information system (HMIS); an early warning reporting and response system (EWARS); and a laboratory-based surveillance program to collect morbidity and mortality data of JE cases. Since May 2004, the WHO-IPD (Programme for Immunization Preventable Disease) has supported Nepal for the active surveillance of JE. The disease has been integrated with the acute flaccid paralysis (AFP) surveillance network and laboratory-based surveillance. Samples from patients suspected of an acute encephalitis syndrome (AES) were collected from 127 sentinel sites for JE diagnosis.

### Data collection and management

For this study, confirmed JE cases (diagnosed using anti-JE IgM antibody capture ELISA), for the period from 2007 to 2015, were collected from the Acute Encephalitis Syndrome (AES) surveillance unit of the WHO/IPD. The data included the number of cases by year, months, district, and age of the patients. The cases were verified with the WHO-IPD fact sheet to check the reliability and consistency of the data, comprising all endemic cases recorded at the sentinel sites in Nepal. Geographical data were collected from the Department of Land Survey and the projected population data (2007 to 2015) were collected from the Department of Health Service, Government of Nepal. The use of these surveillance data for analysis and publications was approved by the Child Health Division, Ministry of Health, Government of Nepal (vide letter no 1427).

### Data analysis

A descriptive data analysis was performed using Microsoft excel 2007 (Microsoft for Windows, Redmond USA). The morbidity rate (number of JE cases per 100,000 population per year) and case fatality rate (number of deaths/number of cases diagnosed per year) were estimated to understand the burden of JE in humans. The seasonal and annual trend of JE cases were also analyzed and represented using graphs. The reported JE cases were integrated with projected population data (2007–2015) to compute yearly-specific incidence for each district. Annualized JE incidence per 100,000 populations at risk were also analyzed. The JE incidence was mapped using an open source Quantum Geographic Information system (QGIS).

A spatial interpolation analysis was performed, using the centroid of each district as a point layer, to estimate a continuous distribution of JE incidence. An inverse distance weighing (IDW) method (Spatial Analyst Tools; ArcGIS™) was used to interpolate JE incidence in humans. The IDW is a moving average or distance-weighted average method and assumes that each interpolation surface should be influenced the most by nearby points and the least by more distant points. The IDW assumes that each measured point has a local influence that diminishes with distance. For example, to predict a value for any unmeasured location, IDW uses the measured values surrounding the prediction location. The resulting interpolation was then displayed as a continuous, graduated color surface of the centroid of each district.

Global spatial autocorrelation analysis was performed using Spatial Analyst extension in ArcGIS10.2.2 software (ESRI, Redlands, CA, United States). First, a contiguity-based spatial weight was constructed for each district by creating a first order rook contiguity weights file to define the spatial relationships using the JE incidence as the variable of interest. Moran's I spatial autocorrelation statistic was then calculated to determine if the JE incidence (JE cases/100,000 population) at the district level was clustered, dispersed, or random. A value of Moran’s I close to 0 implies that JE incidence is distributed randomly in space. A positive value of Moran’s I indicates clustering whilst a value near -1 is indicative of dispersion. The significance of Moran’s I were assessed employing a z-statistic. The spatial correlation at the global level is significant (P = 0.05) when the Z-score is greater than 1.96 or less than -1.96. Since the global spatial autocorrelation was significant (Moran’s Index = 0.39775, Z-score = 5.527, P = <0.001), the spatial distribution of JE incidence at the district level was further investigated for clustering and outliers using a local indicator of spatial association (LISA) Moran’s test. This test detects local spatial autocorrelation (regions where adjacent have similar values) and can also be used as a diagnostic test for outliers in global spatial patterns. The variable of interest in this analysis was the district level JE incidence (JE cases/100,000 population). A rook contiguity weight file to define the spatial relationships using the JE incidence as the variable of interest was used in this analysis. The Local Moran’s Index and the z-score were used to assess the significance of observed spatial correlations and then visualized in the form of LISA cluster map. A high positive Z-score indicates that the surrounding areas have either similarly high JE incidence (High-High) or low JE incidence (Low-Low), while a low negative Z-score indicates a significant (P<0.05) spatial outlier (High-Low) or (Low-High) [17].

## Results

### JE cases and incidence

From 2007 to 2015, 1,823 JE cases (mean of 202 cases/year) in humans were reported nationwide with a cumulative mean incidence of 0.735/100,000 population, ranging between 0.289/100,000 and 1.648/100,000. The case fatality due to JE was 13.8% during 2007 and the trend decreased until 2010 with no record of deaths between 2011 and 2013. The disease was reported again during 2014 and 2015, with an overall case fatality rate of 6.6% during the nine year period (Table 1).

**Table 1:** Total JE cases and case fatality rate in human in Nepal (2007–2015).

Year	No. JE cases	No. deaths	Case fatality rate (%)
2007	442	61	13.8
2008	339	39	11.5
2009	146	9	6.1
2010	197	1	0.6
2011	128	0	0
2012	79	0	0
2013	127	0	0
2014	226	10	4.2
2015	139	1	0.7
Total	1823	121	6.6

### Age distribution of JE cases

The JE cases and fatality were more commonly reported in the age group of 1–14 years when compared to other age groups (Table 2).

**Table 2:** Age distribution of JE cases in human in Nepal (2007–2015).

Age group (year)	JE cases	Deaths (%)
<1year	93	4 (4.32)
1–4	266	17 (6.39)
5–9	447	19 (4.25)
10–14	351	16 (4.55)
15–19	112	7(6.25)
20–24	83	8 (9.6)
>25 year	471	50 (10.6)
Total	1823	121 (6.63)

### Temporal pattern of JE cases

**Fig 1:**
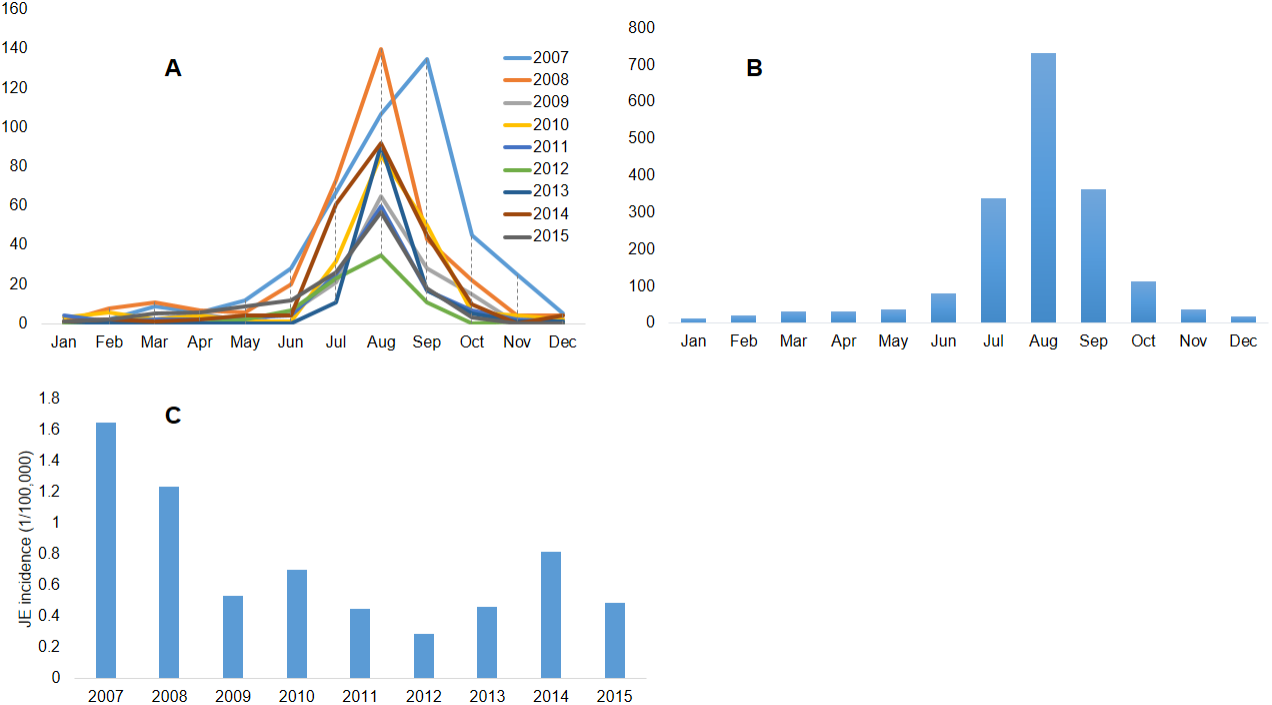
Panel A: Monthly and annual distribution of reported JE cases in human in Nepal (2007–2015), panel B: Monthly cumulative JE cases reported in human in Nepal (2007–2015), panel C: Annual JE incidence (JE cases/100,000 population) in human in Nepal (2007–2015).

The distribution of JE cases in Nepal demonstrates a clear seasonal pattern of occurrence. Although JE cases were reported throughout the year, cases build from June-July, peaked in August and declined by October each year (Fig 1 panels A and B). The highest incidence rate (1.648/100,000) was reported during 2010 and the lowest incidence rate (0.289/100,000) was reported during 2012 (Fig 1 panel C).

### Geographic distribution

**Fig 2:**
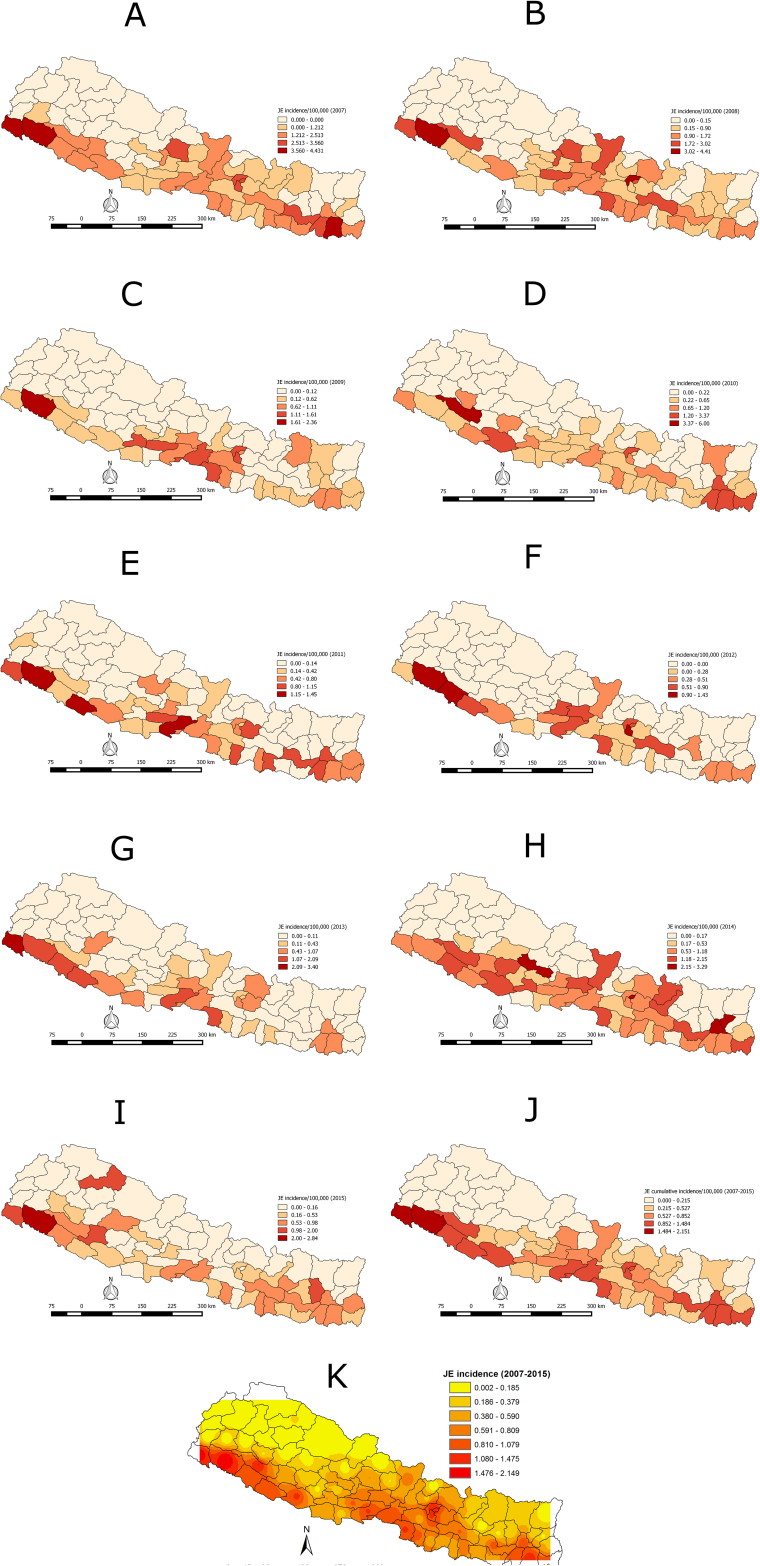
Panels A -K: Spatio-temporal distribution of JE Incidence (JE cases/100,000 population) in human in Nepal (2007–2015) at district level.

**Fig 3:**
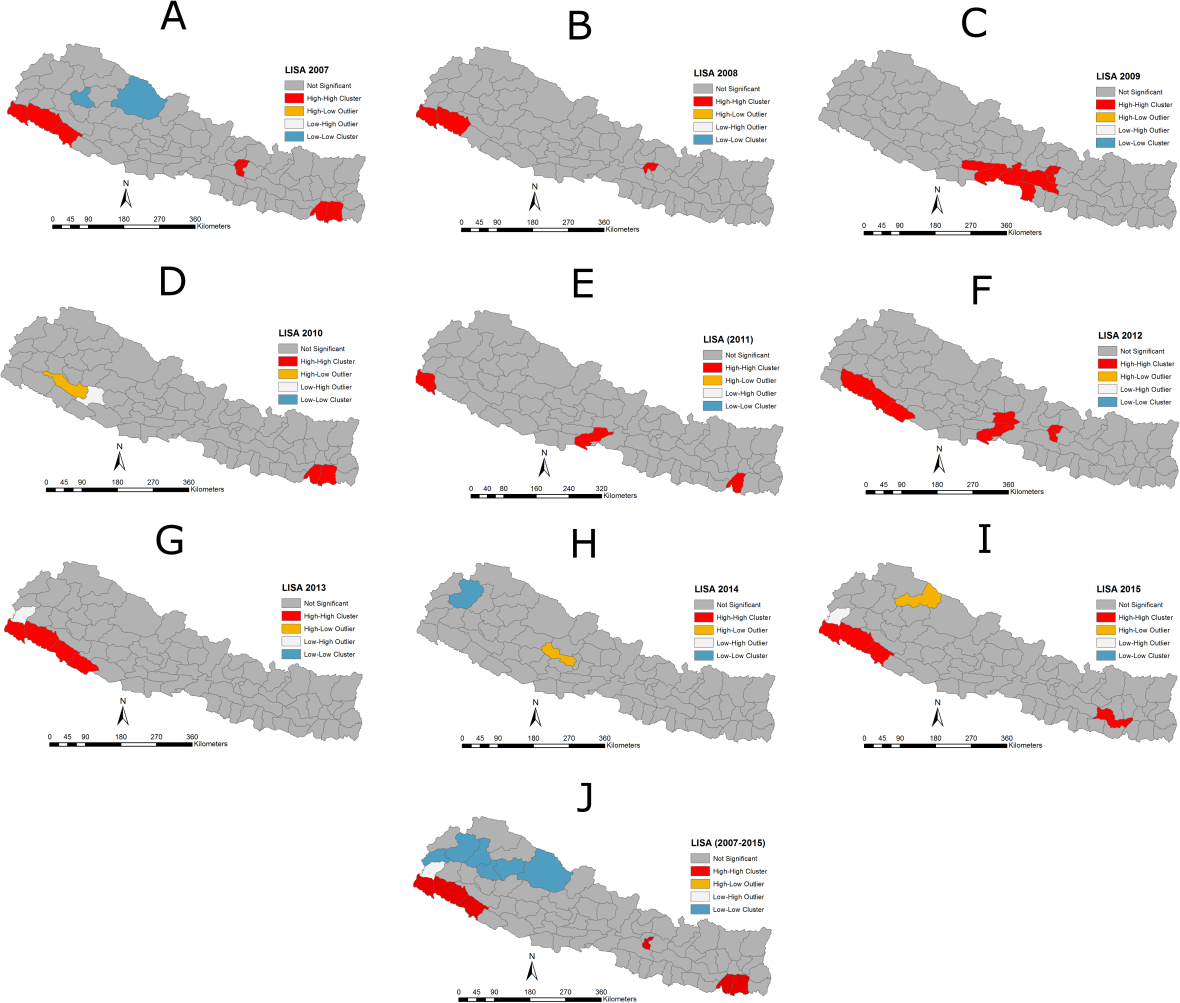
**Panels A -J: Map showing the Local Indicator of Spatial Autocorrelation (LISA) cluster of JE incidences in Nepal.** High-High mean districts/regions with high rate of JE incidence are surrounded by neighboring districts/regions with high rates/incidence of JE with a significant (P<0.05) spatial cluster of high JE incidence. Low-Low indicates a spatial cluster of low JE incidence surrounded by neighboring districts/regions with low JE incidence. High-Low and Low-High are spatial outliers.

During the nine year period (2007–2015), JE was reported in 63 of 75 districts (84%). The cases were reported mostly from the southern Terai low plain area, bordering with India and in Central Nepal. The highest average incidence of 2.15/100,000 was reported in the Kailali district, followed by Kanchanpur (1.76/100,000), Surkhet (1.48/100,000), Morang (1.35/100,000), Kathmandu (1.32/100,000), Bhaktapur (1.27/100,000), Dang (1.16/100,000), Lalitpur (1.13/100,000), Parsa (1.13/100,000), Nawalparasi (1.11/100,000), Sunsari (1.067/100,000), Banke (1.05/100,000) and Bardiya (1.00/100,000). The lowest incidence was reported in the Achham district (0.04/100,000) (Fig 2 panels A -K). Twelve districts have not reported JE cases. There was strong clustering of JE at the global level (Moran’s I = 0.39775, Z-score = 5.527, P = <0.001). The LISA analysis indicated high-high clustering of JE incidence in the southeast and southwest Terai region, and in Central Nepal (Fig 3 panels A-J).

## Discussion

This study demonstrates that JE incidence is not distributed homogeneously in Nepal. Cases were clustered mostly in the southern Terai or low plain land. This might be due to high vector density, high intensity of cultivated paddy fields and favorable climatic factors for the JE vector. Terai is the grain basket of Nepal with high paddy cultivation and animal farming, including swine and birds. This ecological region has been reported as a JE prone area in the previous studies [11, 18, 19]. Therefore, preventive and control measures should be adopted before the outbreak period in this high risk zone. Although there has been a slight decline of JE cases in Nepal in the recent past, geographical expansion into hilly and mountain regions of the country has been documented between 2014 and 2015 (Fig 2). This might be associated with changes in socio-ecological determinants, including changing agriculture practices and land use patterns, climate change, expansion of pig farming and other anthropologic determinants. For example, Tibet, located in the Qinghai-Tibet Plateau of the western People’s Republic of China, has been recognized previously as free of JE because of high elevation. However, this region has recently demonstrated the emergence of JE virus in mosquitoes, humans and pigs, indicating global expansion of JEV into non-endemic areas [20]. Factors such as global warming, increased pig farming, and increased tourism and transportation may have contributed to the emergence of JE in Tibet. Among various driving predictors for JEV, climatic variation is regarded as a key factor. Change in both temperature and precipitation are capable of affecting JEV transmission [21–24]. Studies of JE epidemiology in Japan, Nepal, China and Taiwan demonstrated that transmission was associated with high temperature and low precipitation [25–28]. Nepal is one of the world’s vulnerable countries with respect to climate change. The increasing average annual temperatures in the hill and mountain regions of the country might have provided conducive environments for the survival and adaptability for JE vectors (*C*. *tritaeniorrhynchus*), thus favoring geographical expansion of JE in Nepal [29]. In addition, other climate-sensitive vector-borne diseases, including malaria, lymphatic filariasis, visceral leishmaniasis and dengue, have been reported in the hilly and mountain regions of Nepal, which were previously considered as non-endemic, indicating the adaptation of vectors in non-endemic regions [30, 31]. The study also detected anti-JE virus antibodies in swine populations sampled from four high-altitude mountain districts of Nepal indicating circulation of JE virus, where human infections were documented [32]. Similarly, a study conducted by Impoinvil et al. (2011) demonstrated JE clusters shifting to hilly and mountain regions of Nepal [26]. Therefore, a more comprehensive surveillance system should be designed and implemented based on a One Health approach for the early detection, preparedness and monitoring of the JE situation in Nepal.

The JE incidence in Nepal demonstrates a clear seasonal pattern. More cases were reported during the rainy season during June-July, peaking in August and declining by October (Fig 1 panels A and B). Similar patterns were also observed in past epidemics. The seasonal pattern of JE in Nepal is also in agreement with findings from other studies occurring commonly during the monsoon season [2, 33, 34]. Understanding the seasonality of JE incidence in Nepal provides important information for implementing general public health education about the risk of JE outbreaks and the importance of taking precautionary measures during the monsoon season. In addition, given the seasonality of the disease, vaccination against JEV can also be planned and completed at least one month prior to the onset of the monsoon season to ensure that vaccinated individuals develop sufficient JEV immunity.

The JE case detection in Nepal shows a declining trend over the years, which may be due to effective intervention programs, including immunization, enhanced surveillance and case management, which are priority intervention measures of JE control in Nepal. In other countries, such as Japan, the Republic of Korea, Brunei, Australia, and Malaysia, a comprehensive immunization program has significantly reduced JE cases [35]. The Government of Nepal has conducted mass immunization campaigns in six districts with the highest JE burden during 2006 and expanded the campaign to 20 (83%) of 24 Terai and 3 (9%) of 35 hill districts during 2009, with an estimated campaign coverage rate of 94%. The post-campaign JE incidence rate of 1.3 per 100,000 population was 72% lower than expected if no campaigns were conducted [13]. This phase-wise immunization program has helped to reduce drastically the JE incidence in Nepal. However, a slight inclining trend of JE was observed since 2012, which might be due to various socio-environmental factors. For instance, a cross-sectional study, conducted in four districts of Nepal (Rupandehi, Kapilvastu, Morang, Kathmandu valley), indicated socio-cultural factors, such as literacy, gender, and cultural practices associated with poor farmers’ knowledge, attitude and practices for JE control. The JE vaccine uptake was non-existent and mosquitoes control steps were applied inconsistently across all four districts [36]. Furthermore, the lower rate of JE incidence in remote places (e.g., remote district of Achham) may be associated with socio-ecological factors, lack of healthcare facilities, healthcare accessibility, poor healthcare seeking behavior of people and weakness in the effectiveness of the surveillance system, thus resulting in an under reporting of JE cases. Also, due to remote locations and fewer healthcare facilities, those areas might not have been included in the sentinel surveillance system.

The morbidity rate due to JE is high for the 1–14 year-age group in Nepal. In the absence of immunization programs, persistence of infection in the area helps in the development of naturally-acquired immunity in older age groups when compared to children, thus making them into a high risk group for the disease. Prior to the implementation of vaccination programs, children under 15 years were considered as the high-risk group for JE in China, India, Thailand, and Sri Lanka, whereas all age groups are considered at risk of JE infection in Burma and Nepal [6]. In some countries, including Japan and Taiwan where there is wide utilization of vaccines and other control measures, a modest shift in age distribution towards adults has been observed [37]. Since the cases have been reported in the young as well as adults, a national policy on immunization needs to be modified and also be expanded to the hilly and mountainous regions, focusing upon all age groups. Owing to only sporadic JE cases in the mountainous region of Nepal, both the children and adult populations may not have been exposed to the virus, unlike in the Terai region, making them susceptible to infection. Implementation of disease monitoring in swine would also help to identify high risk zones for vaccination. In addition, molecular analyses should be conducted to study the origin of JE virus and mosquitoes. Since ardeid birds are reservoirs of JEV, monitoring the sero prevalence of JEV in birds from low and highlands may provide information about the relative extent of JEV transmission in these two areas. This will help to bridge the gap between medical, veterinary and wildlife sectors for more effective disease control.
